# Intravital mesoscale optical imaging: challenges, techniques, and future perspectives

**DOI:** 10.52601/bpr.2025.250015

**Published:** 2026-02-28

**Authors:** Mingrui Wang, Jiamin Wu, Qionghai Dai

**Affiliations:** 1 Tsinghua Shenzhen International Graduate School, Tsinghua University, Shenzhen 518071, Guangdong, China; 2 Department of Automation, Tsinghua University, Beijing 100084, China; 3 Institute for Brain and Cognitive Sciences, Tsinghua University, Beijing 100084, China

**Keywords:** Intravital microscope, Mesoscale optical imaging, Space bandwidth product, Cellular dynamics, Tomography, Optical aberration

## Abstract

Intravital mesoscale imaging plays a crucial role in bridging the gap between cellular and organ-level investigations by enabling high-resolution visualization across large fields of view. Continuous advancements in optical microscopy have significantly improved imaging performance, yet fundamental challenges remain. Effective intravital mesoscale imaging requires a balance between spatial resolution, imaging speed, field of view, and while overcoming limitations such as scattering, aberrations, phototoxicity and photobleaching. This review summarizes key challenges in achieving high-performance intravital mesoscale optical imaging and provides an overview of advanced optical imaging techniques, including wide field, laser scanning, as well as computational imaging approaches. Despite these advancements, further improvements are necessary to address existing limitations and unlock new possibilities. Future developments will focus on enhancing imaging depth, further improving space bandwidth products, and integrating computational methods for real-time processing and large-scale data analysis, further advancing mesoscale imaging for biological research.

## INTRODUCTION

**Figure 1:**
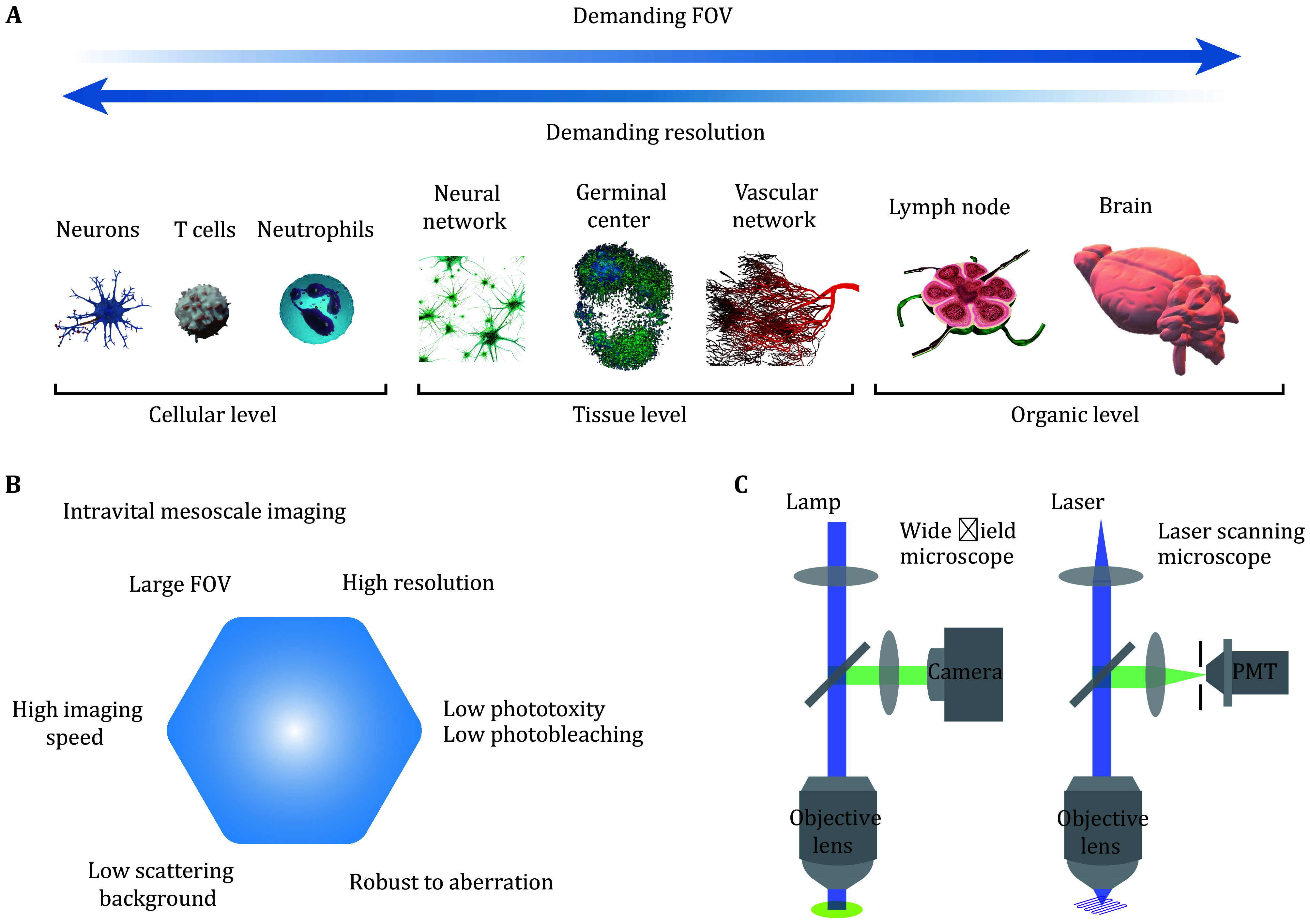
**A** Illustration of biological frameworks which inherently operate across multiple scales, ranging from the cellular to the organ level. **B** Basic requirements of intravital mesoscope imaging. **C** Comparison of the framework between wide field imaging and laser scanning imaging

Visualizing cellular dynamics clearly at the mesoscale level is necessary for understanding various physiological processes. Many advanced optical microscopes for intravital imaging provide access to visualizing and understanding biological systems at high spatiotemporal resolution, facilitated by the development of optical design (Bennett 1943; Brady and Hagen 2009; McConnell *et al.* 2016; Negrean and Mansvelder 2014; Yan *et al.* 2017), mechanical engineering (Csencsics *et al.* 2020; Grandhe and Bandopadyay, 2023), and computational processing (de Haan *et al.* 2020; Rivenson *et al.* 2017; Wang *et al.* 2019; Weigert *et al.* 2018). Various imaging strategies have been developed, each with distinct advantages depending on the sample characteristics and imaging requirements. Wide-field imaging provides a rapid approach for planar imaging, enabling high temporal resolution even across large imaging fields of view (Werley *et al.* 2017). This technique is commonly used for thin samples and transparent specimens with fast dynamic processes. Laser scanning microscopes such as traditional two-photon microscopes (Denk *et al.* 1990), often provide clear imaging in scattering living samples due to their ability to physically reject out-of-focus signals. With the development of imaging techniques, microscopy has gradually advanced toward mesoscale and become more suitable for intravital imaging. Among the pioneering works in this field, Karel Svoboda and his colleague developed the two-photon mesoscope, enabling large-scale neural circuit imaging with subcellular resolution (Sofroniew *et al.* 2016). Gail McConnell’s group introduced the Mesolens, which combines a large field of view with a high numerical aperture for volumetric imaging (McConnell *et al.* 2016). Our lab has also been a key leader in the invention of intravital mesoscale imaging with one of the pioneering works of the RUSH (real-time, ultra-large-scale, high-resolution) system which can be traced back to 2013, when we undertook a major scientific instrumentation project funded by the National Natural Science Foundation of China (Fan *et al.* 2019). All of these breakthroughs have laid the foundation for intravital mesoscope imaging, pushing the boundaries of mesoscale biological research. These systems often maintain high cellular spatial resolution while expanding the field of view (Fig. 1A) and achieving fast imaging speeds to capture dynamic cellular activities. Additionally, for complex intravital environments, it is crucial to address challenges such as scattering background and sample-induced aberrations (Pittet and Weissleder 2011). Moreover, to enable long-term imaging, considerations must be given to laser-induced photobleaching of fluorescent proteins (Klonis *et al.* 2002) and photodamage to biological samples (Fig. 1B) (Hopt and Neher 2001; Magidson and Khodjakov 2013). Therefore, mesoscale intravital imaging requires systemic improvement of all aspects of traditional optical microscopy, including the field of view, resolution, 3D imaging speed, low phototoxicity, aberration robustness, and high fidelity against background fluorescence, leading to grand challenges in the optics field.

This review will introduce intravital mesoscope imaging methods. First, the challenges associated with achieving high-performance intravital mesoscale imaging will be discussed. Then, advanced mesoscale imaging techniques will be presented, highlighting their principles, key characteristics, and suitable applications. Finally, the review will explore the future potentials of intravital mesoscope, discussing the remaining limitations and key challenges that need to be addressed for further enhancement, analysis and applications.

## CHALLENGES OF INTRAVITAL MESOSCALE IMAGING

**Figure 2:**
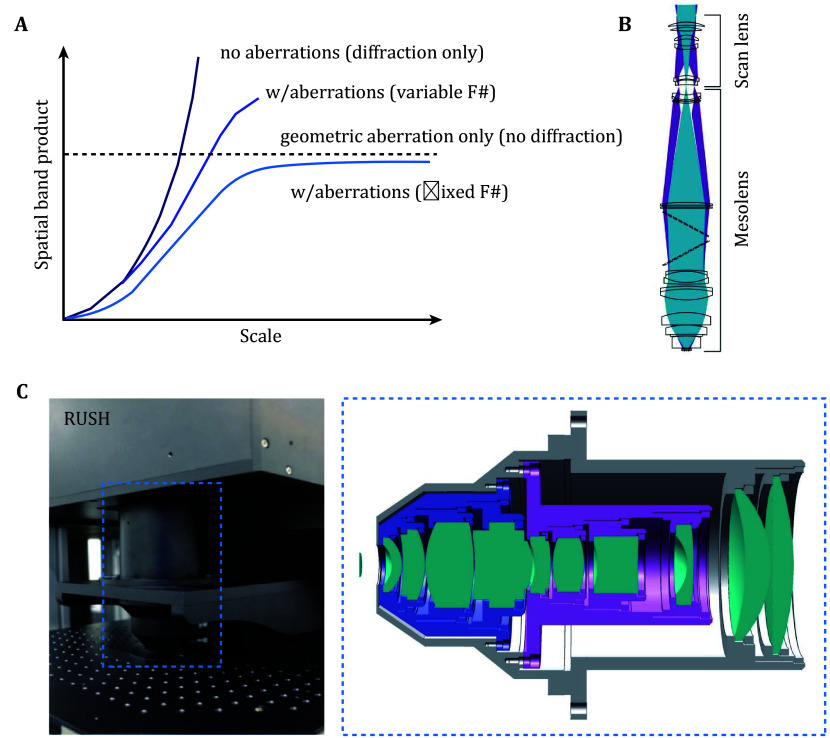
**A** Space-bandwidth product of an optical system as a function of scale for no aberrations (top), with aberration (variable F#), no diffraction (dashed line) and with aberration (fixed F#) (bottom) (Lohmann 1989). **B** Optical design of mesolens objective lens (McConnell *et al.* 2016). **C** Optical design of objective lens of RUSH system. Left, photograph of the objective lens. Right, optical design details (Fan *et al.* 2019)

To capture the entire landscape of biological systems, microscopes need to be equipped with a large field of view (FOV), typically ranging from several millimeters to even centimeter scale, while maintaining high resolution to achieve cellular-level details. The amount of information provided by optical imaging techniques is quantified as the space bandwidth product (SBP) (Lohmann *et al*. 1996; Mendlovic *et al*. 1997), with a higher SBP indicating greater information acquisition. However, two key factors limit the increase of SBP: first, the sampling rate of the imaging system (Orth and Crozier 2013); second, the performance of optical access (Fig. 2A), as the resolution is constrained by optical diffraction, and system aberrations tend to larger as the FOV expands (Lohmann 1989).

To effectively record cellular dynamics in living samples – such as neutrophil migration within blood vessels (Lerman and Kim 2015; Salvermoser *et al*. 2018; Xu *et al*. 2022), calcium signaling (Augustine *et al*. 2003; Berridge 1998) or voltage fluctuations representing neuronal activity (Peterka *et al*. 2011) – imaging systems must achieve high-speed acquisition, typically ranging from one to hundreds of frames per second (fps). This combination of a large FOV, high resolution, and fast imaging speed is critical for capturing the complex and dynamic processes occurring in biological systems.

Most living samples are composed of highly scattering tissues, creating complex imaging conditions that hinder the detection of signals, especially from deeper layers (Cheng *et al*. 2020). The scattering not only limits imaging depth but also generates substantial background noise, in contrast to the clear environment of transparent *ex vivo* samples, thereby significantly reducing the signal-to-background ratio (SBR) (Zhang *et al*. 2021a). Laser scanning microscopy addresses this challenge by utilizing point-scanning techniques and effectively rejecting out-of-focus light, enabling the acquisition of clear cellular images even in scattering environments (Davidovits and Egger 1971; Denk *et al*. 1990; Minsky 1988; Otsu *et al*. 2008). Besides, biological samples often introduce considerable optical aberrations due to refractive index mismatches. These aberrations further degrade image quality, posing additional challenges for high-resolution intravital imaging (Schwertner *et al*. 2004).

A wide range of biological processes, such as tumor metastasis (Condeelis and Weissleder 2010; Khanna and Hunter 2005), germinal center formation in lymph nodes (Gonzalez-Figueroa *et al*. 2021; MacLennan 1994; Victora *et al*. 2010) and other immune response after organ injury (An *et al.* 2025) require extended periods to complete, ranging from several hours to even days, often accompanied by complex and dynamic changes. However, prolonged exposure to light introduces phototoxicity effects (Gottschalk *et al*. 2015), leading to cellular damage or even cell death. Photobleaching is another critical concern. Fluorescent proteins or dyes, commonly used to enhance imaging contrast (Prasher 1995; Shimomura *et al*. 1962), gradually lose their fluorescence under continuous illumination, thereby limiting the duration and quality of imaging sessions. Currently, the intravital mesoscope must carefully balance these factors. Therefore, optimizing imaging systems to minimize light-induced damage while maintaining sufficient image quality is a key challenge in long-term intravital imaging.

Nowadays, with the advancement of imaging technologies, various mesoscale imaging methods have been developed to address the aforementioned challenges, making them more suitable for intravital applications. To better illustrate the capabilities of different mesoscale imaging techniques, Table 1 summarizes key performance metrics, including spatial resolution, temporal resolution, and FOV. This comparison provides insights into the strengths of each imaging system to help identify the most suitable technique for specific intravital applications. This review will delve into the details of these mesoscale imaging techniques, discussing their technological developments, optimal application scenarios, distinctive characteristics, and the key methodologies that underpin their performance.

**Table 1:** Comparison of representative intravital mesoscale imaging techniques in terms of their spatial resolution, temporal resolution, and FOV across different modalities

Year	Name of techniques	Reference	Basic parameters				Simple description
			FOV (mm)	Resolution (μm)	Imaging speed (fps)	Effective pixel (voxel) acquisition rate (kHz)	
2016	2p-RAM	Sofroniew *et al.* 2016	Φ5 × 1	Lateral: 0.66; Axial: 4.09	0.7	7.7 × 10^3^	Two photon imaging with subcellular resolution
2016	Confocal Mesolens	McConnell *et al.* 2016	Φ6 × 3	Lateral: 0.8;Axial: 8	0.005	3.3 × 10^2^	Confocal imaging
2016	Treapn2p	Stirman *et al.* 2016	Φ3.5	Lateral: 1.2;Axial: 12	0.1	6.7 × 10^2^	Two photon imaging with temporal multiplexing
2017	Firefly	Werley *et al.* 2017	Φ6	Lateral: 7	100	3.4 × 10^4^	Wide field imaging
2019	RUSH	Fan *et al.* 2019	12 × 10	Lateral: 0.8	30	5.6 × 10^6^	Wide field imaging, 35 sCMOS for detection
2021	FASHIO-2PM	Ota *et al.* 2021	3 × 3	Lateral: 1.61; Axial: 7.07	7.5	5.9 × 10^3^	Two photon imaging
2021	Quadroscope	Clough *et al.* 2021	Φ4.8	Lateral: 0.91; Axial: 10.5	30	5.1 × 10^3^	Two photon imaging with temporal multiplexing
2021	Diesel2p	Yu *et al.* 2021	Φ5	Lateral: 1; Axial: 8	3.85	1.7 × 10^4^	Two photon imaging with temporal multiplexing and AO
2021	LBM	Demas *et al.* 2021	5.4 × 6 × 0.5	Lateral: ~5	2	3.9 × 10^4^	Two photon imaging with axially separated and temporally distinct foci
2022	MINI2P	Zong *et al.* 2022	5 × 5 × 0.16	Lateral: ~1.2; Axial: ~14	15 (single FOV)	2.9 × 10^4^	Miniature head-mounted 2p mesoscope
2023	3D-RAPID	Zhou *et al.* 2023	12 × 10.8 × 0.9	Lateral: 25	15	2.8 × 10^4^	Wide field imaging, 54 sCMOS for detection
2023	Mesoscopic OPM	Daetwyler *et al.* 2023	3.7 × 1.5 × 1	Lateral: 2.3; Axial: 9.2	5	5.7 × 10^5^	Single objective lens light sheet imaging
2024	RA-WiFi	Shi *et al.* 2024	12.8 × 12.8	Lateral: 2.18	2	6.8 × 10^4^	Random access wide field imaging with AO correction
2024	SOMM	Zhang *et al.* 2024b	3.6 × 3.6 × 0.3	Lateral: 4	16	1.3 × 10^4^	Miniature mesoscope
2024	RUSH3D	Zhang *et al.* 2024a	8 × 6 × 0.4	Lateral: 2.6; Axial: 6	20	7.4 × 10^7^	Scanning light field imaging, DAO for aberration correction

## TECHNIQUES OF INTRAVITAL MESOSCALE IMAGING

Current imaging techniques for intravital mesoscale imaging can be broadly categorized into two approaches: wide field imaging and laser scanning imaging (Fig. 1C) (Schneckenburger and Richter 2021). Besides, advancements in computational imaging have further enhanced both imaging approaches (Dong *et al.* 2023; Mait *et al.* 2018; Zhao *et al.* 2023), offering solutions to overcome inherent trade-offs between resolution, speed, and imaging depth.

Wide field imaging involves uniformly illuminating the entire observation area at once, followed by capturing the image using a fast photon array detector, such as a high-speed CMOS, CCD or sCMOS camera. This approach allows for rapid acquisition of large-area images, making it highly efficient for visualizing extensive regions of tissues or organs (Werley *et al*. 2017; Zheng *et al*. 2024). However, wide field imaging is often hindered by out-of-focus background signals or scattering from within the sample, which can reduce image contrast and obscure fine details.

In contrast, laser scanning methods, such as confocal microscopy (Davidovits and Egger 1971; Sheppard and Choudhury 1977) or nonlinear techniques like two-photon excitation microscopy (Denk *et al*. 1990; Helmchen and Denk 2005), operate by sequentially exciting and detecting signals from individual points within the biological sample. This point-by-point scanning approach effectively suppresses background fluorescence and scattered light, yielding higher contrast images with improved optical sectioning. Nevertheless, the speed of laser scanning methods is inherently limited due to the point-by-point data acquisition. This limitation becomes particularly pronounced when imaging larger fields of view, as the increased number of sampling points significantly extends the acquisition time.

The trade-offs between wide field and laser scanning methods highlight the challenges of achieving both high-speed and high-contrast imaging intravitally, especially when imaging at the mesoscale level. Based on their imaging principles and characteristics, these techniques are suited to different application scenarios (Oleksiievets *et al*. 2022; Swedlow *et al*. 2002). With ongoing advancements in imaging technology, both approaches are continuously evolving to enhance their strengths while addressing their inherent limitations. For instance, innovations in wide field imaging aim to mitigate background and scattering effects through techniques like structured illumination (Gustafsson 2000; Gustafsson *et al*. 2008) or light-sheet illumination (Huisken *et al*. 2004). Similarly, laser scanning methods are being improved with faster scanning mechanisms, such as resonant scanning or multi-point excitation with parallelized beam paths (Nikolenko *et al*. 2008; Otomo *et al*. 2015; Yang and Yuste 2018) or temporal focusing (Vaziri and Shank 2010), to overcome speed limitations. These developments enable both wide field and laser scanning techniques to better meet the demands of intravital mesoscale imaging, making them increasingly versatile for current applications.

Beyond advancements in optical system, computational imaging has emerged as a powerful approach to enhance both wide field and laser scanning techniques. Techniques such as light-field microscopy (Prevedel *et al.* 2014), scanning light field microscopy (Lu *et al.* 2025; Wu *et al.* 2021; Zhang *et al.* 2024a), compressive sensing-based reconstruction (Wen *et al.* 2019), and deep-learning-assisted tomography (Wang *et al.* 2021) have demonstrated remarkable potential in overcoming trade-offs between resolution, speed and FOV. Computational imaging approaches can also compensate for scattering effects and aberrations (Zhang *et al.* 2024a), making them particularly valuable for intravital mesoscale imaging applications.

### Wide field imaging

Wide field imaging usually illuminates and captures the entire field of view simultaneously. Wide field framework is easy to set up and widely applicable across various scenarios (Holtmaat *et al*. 2009; Xiao *et al*. 2024; Yang *et al*. 2010; Zheng *et al*. 2013, 2024). It is not only utilized in table-set microscopes for mesoscale imaging but also adapted for miniaturized devices, such as head-mounted microscopes (de Groot *et al.* 2020; Guo *et al.* 2023; Rynes *et al.* 2021; Zhang *et al.* 2024b), enabling large FOV imaging in freely behaving animals.

For mesoscale microscope imaging, we often need higher SBP to cover more information (Park *et al*. 2021). In practice, two key factors limit the SBP, the sampling rate of the imaging sensor and system optical diffraction aberration. Nowadays, advanced high-resolution CMOS sensors can accommodate a large number of pixels, and the use of camera arrays (Fan *et al.* 2019; Zhou *et al.* 2023) further enhances spatial digital sampling rates. More commercial and customized objective lenses (Kim *et al*. 2016; McConnell *et al*. 2016) are being developed to expand the FOV and minimize optical aberrations, such as astigmatism and field curvature, particularly at the edges of the imaging field (Welford 2017). McConnell *et al*. developed a complex mesoscale lens (Fig. 2B) that comprised 15 optical elements for aberration corrections (McConnell *et al*. 2016). Fan *et al*. developed the RUSH system, which features a centimeter-scale field of view and a spatial resolution of 0.8 micrometers (Fan *et al*. 2019). The system employs a custom-designed large lens that effectively corrects aberrations across a wide field (Fig. 2C). Additionally, it integrates a camera array with 35 cameras for simultaneous imaging, significantly enhancing spatial sampling. Notably, it is the world’s first real-time gigapixel fluorescence microscope for intravital imaging and remains among the leading systems in data throughput. A three-dimensional (3D) parallelized computational wide field mesoscope implements 54 cameras for large scale and not only increases the spatial sampling rate (Zhou *et al*. 2023), but also captures information from different views for 3D reconstruction. These advancements have significantly improved the imaging system’s SBP, enabling rapid acquisition of detailed information across a large FOV.

However, a large FOV also introduces complex challenges, such as defocus aberrations caused by curved surfaces, blank regions that record unnecessary data, and more complex higher-order aberrations induced by mismatched refractive indices (Potsaid *et al*. 2005). Several systems implement digital micromirror devices (DMD) for structure illumination, a series of different thicknesses of glass (Xie *et al*. 2024) or electrically tunable lens (ETL) (Shi *et al*. 2024) and deformable mirrors for refocusing specific regions. For example, Shi *et al*. developed RA-WiFi (random-access wide field) mesoscope using a commercial objective lens, extending the working distance to achieve a larger FOV enabling imaging on a scale of one square centimeter and implementing a scanning mirror to record a specific region, which is illuminated by the reflection of DMD, and use ETL and deformable mirror for the aberration correction mainly for defocus (Shi *et al*. 2024). These methods have made imaging systems more flexible, enhancing the proportion of useful information and making them better suited for more complex intravital imaging environments.

**Figure 3:**
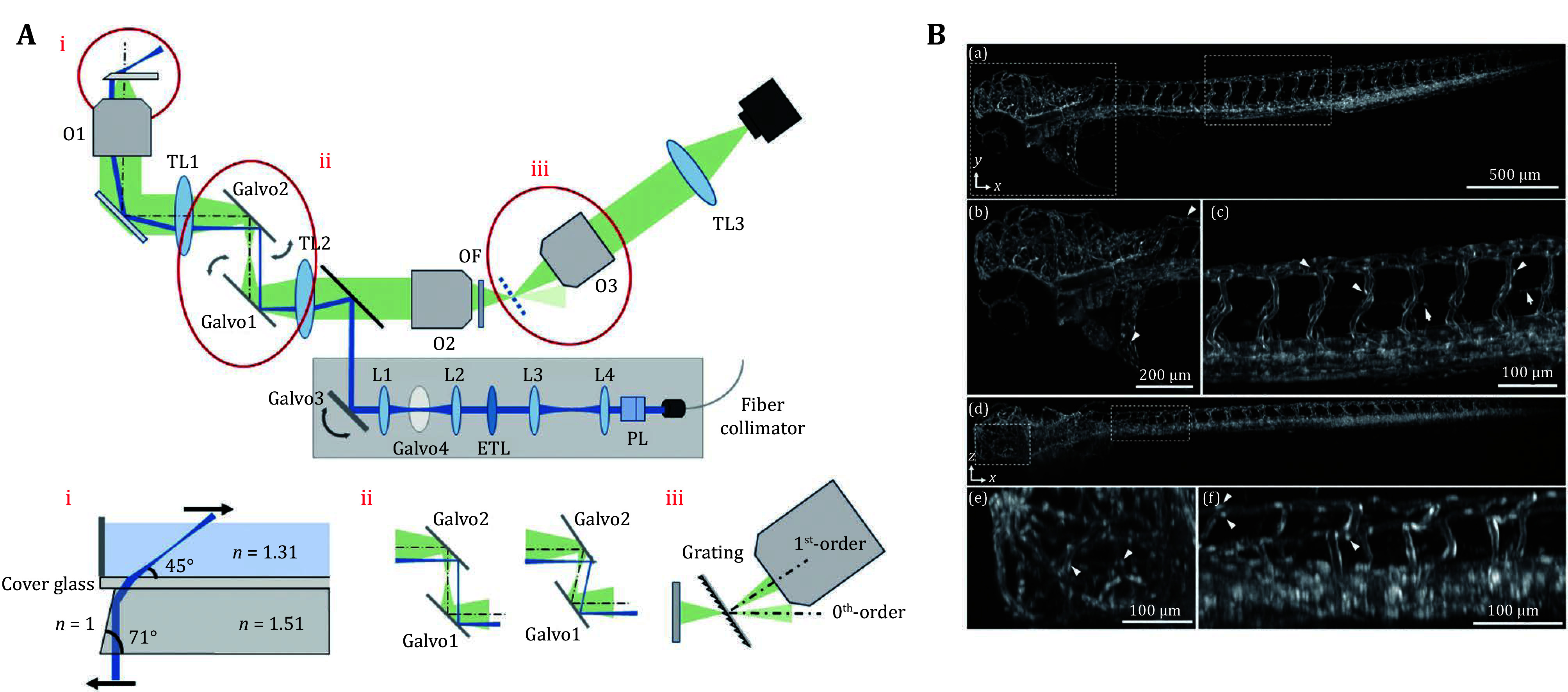
**A** Schematic setup of the mesoscopic oblique plane microscope. O1–O3, primary, secondary, and tertiary objectives; TL1–TL3, primary, secondary, and tertiary tube lenses; OF, optical flat. Inset (i) shows detail of the microprism that reflects the light sheet into the sample. Inset (ii) shows the working principle of the image space scanning. Inset (iii) shows how the blazed diffraction grating diffracts the first order towards the primary objective. **B** Imaging of zebrafish vasculature. Fluorescently labeled vasculature, in a three days post fertilization (dpf) zebrafish larva, as imaged with our mesoscopic OPM. (a) *x*-*y* maximum intensity projection of the entire zebrafish with (b, c) magnified views (head and tail vasculature) of the boxed regions in (a). Arrowheads indicate selected endothelial nuclei, and arrows point to parachordal lymphangioblasts. (d) *x*-*z* maximum intensity with (e, f) *x*-*z* maximum intensity projected magnified views (head and tail vasculature) of the boxed regions in (d). Arrowheads indicate selected endothelial nuclei (Daetwyler *et al.* 2023)

Epi-illumination in wide field microscopy is convenient for system setup and sample placement during experiments, but the out-of-focus background can significantly reduce the SBR. Light-sheet microscopy typically illuminates the focal plane from a direction orthogonal to the imaging objective, effectively reducing the fluorescence background (Huisken *et al*. 2004). By scanning either the objective lens or the sample stage, it enables the reconstruction of a 3D image of the specimen. However, the mechanical scanning of the objective lens and sample stage limits imaging speed, making this approach more commonly used for imaging cleared tissues. Advanced imaging systems now incorporate multi-plane excitation (Ren *et al*. 2020), extended depth of field (DOF) (Tomer *et al*. 2015), and galvo-mirror-scanned light sheets to increase 3D imaging speed for intravital applications, enabling the capture of cellular dynamics, such as recording neuronal activity across an entire zebrafish at 5 Hz (Tomer *et al*. 2015). To eliminate the lateral constraints imposed by the illumination objective lens, Dunsby introduced an approach where an oblique light sheet is emitted from the edge of the imaging objective, enabling single-objective light-sheet microscopy (Dunsby 2008). These systems typically require two objective lenses for 1:1 imaging with minimal aberration, allowing the secondary objective to capture the illuminated plane from a perpendicular angle. To image a living sample at video rate, the light-sheet scanning galvo-mirror is positioned at the back focal plane, ensuring that the image plane remains stationary during acquisition (Bouchard *et al*. 2015; Kumar *et al*. 2018; Kumar and Kozorovitskiy 2019; Voleti *et al*. 2019). To achieve mesoscale imaging with a low-NA objective lens, several methods incorporate diffraction gratings (Hoffmann and Judkewitz 2019), blaze gratings (Hoffmann *et al*. 2023; Shao *et al*. 2022), or high-refractive-index media (Daetwyler *et al*. 2023) to enhance light collection efficiency in single-objective light-sheet microscopy (Figs. 3A and 3B). These methods enable higher SBR 3D mesoscale imaging at video rate for commonly used model organisms. For example, they have been applied to imaging transparent samples, capturing neuronal activity in *C. elegans* (Voleti *et al*. 2019) and zebrafish (Daetwyler *et al*. 2023), and even visualizing cellular dynamic processes in the scattering tissue of the mouse cortex (Bouchard *et al.* 2015; Wang *et al.* 2023).

Overall, wide field imaging techniques, including advanced light-sheet microscopy, have significantly improved the SBP, speed and SBR for mesoscale intravital imaging. These methods enable high-speed, large-scale imaging across various biological models typically with simple optical setups, from transparent organisms to scattering tissues. However, despite these advancements, wide field approaches still face challenges such as background fluorescence and optical aberrations, particularly in deep-tissue imaging. To address these limitations, laser scanning mesoscope offers an alternative strategy with improved optical sectioning and background suppression.

### Laser scanning imaging

Compared to wide field imaging techniques, most laser scanning methods excite a single point and detect the corresponding signal using a single photodetector, such as a photomultiplier tube (PMT). Typically, confocal microscopy employs a pinhole at the conjugate plane to reject out-of-focus fluorescence signals (Davidovits and Egger 1971). In contrast, multiphoton microscopy leverages nonlinear optical effects to generate effective excitation only at the specific focal point (Helmchen and Denk 2005; Horton *et al*. 2013).

The imaging speed of confocal and multiphoton microscopy is often limited by the raster scanning process. To overcome this limitation, several scanning strategies have been developed in confocal microscopy to enable multi-point acquisition, such as line-scan confocal (Im *et al*. 2005) and spinning disk confocal (Lchihara *et al*. 1996), which significantly enhance the ability to capture fast cellular dynamics. However, these methods are still limited in imaging performance due to the influence of scattering media, making them typically suitable for transparent or semi-transparent samples such as *C. elegans* and zebrafish. To track the movement of samples over large areas, a low-magnification imaging system is implemented to monitor sample motion and dynamically adjust the stage, ensuring that the imaging target remains within the field of view of the confocal microscope at cellular resolution (Bai *et al*. 2024; Faumont *et al*. 2011; Zhang *et al*. 2021c). This approach effectively expands the field of view of confocal microscopy while maintaining high-resolution imaging.

Compared to confocal microscopy, two-photon microscopy offers superior anti-scattering capabilities, allowing for deeper tissue penetration. Additionally, it induces lower phototoxicity, making it well-suited for mesoscale imaging of organs in many mammalian models. For example, an adaptive brightness modulation system was integrated into a multiphoton microscope, enabling high-quality 3D dynamic imaging of entire lymph nodes (Pinkard *et al*. 2021). The Bessel focus module was incorporated into two-photon imaging, achieving mesoscale volumetric synaptic imaging of the mouse cortex (Lu *et al*. 2020). Several works have miniaturized two-photon microscopes to record neuronal activity in the brains of freely moving rodents (Ziv and Ghosh 2015; Zong *et al*. 2022). Among them, Zong *et al*. optimized the design and developed the MINI2P system, which is capable of recording large-scale, multi-layer neuronal activity, capturing signals from up to 10,000 neurons in the same animal (Zong *et al*. 2022).

Advances in two-photon microscopy are increasingly focused on achieving large FOV, high-speed, multi-depth plane intravital imaging (Demas *et al.* 2021; Sofroniew *et al*. 2016; Stirman *et al.* 2016). To simultaneously expand the imaging depth and FOV while maintaining high spatial and temporal resolution as well as a high signal-to-noise ratio (SNR), it is essential to overcome two main challenges: optimizing the optical system and increasing the speed of sampling (Ji *et al*. 2016).

Typically, the objective lens and scan engine need to be optimized to increase the field of view while maintaining a high scanning speed and fully utilizing the numerical aperture (NA) of the objective lens, which is crucial to ensure high resolution and SNR. Several works use commercial or customized optical elements to design and set up the two-photon mesoscale imaging system (Ota *et al.* 2021; Tsai *et al.* 2015; Yu *et al.* 2024). For example, Tsai *et al*. developed a serial scanning engine combined with a 0.28 NA commercial objective that recorded across a centimeter-scale FOV (Tsai *et al*. 2015); Ota *et al*. developed FASHIO-2PM, featuring an optimized resonant scanning system and a customized large objective lens with low magnification and high NA (NA = 0.8), enables the recording of 16,000 neurons at 7.5 Hz from a 9 mm^2^ contiguous imaging plane (Ota *et al*. 2021). Yu *et al*. developed the Cousa objective, a long-working distance (20 mm) air objective capable of covering an area of over 4 mm^2^, making it highly suitable for intravital mouse brain imaging (Yu *et al.* 2024).

To improve the speed of sampling, several strategies can be employed. First, utilizing faster scanning tools can significantly enhance imaging speed. For instance, inertia-free acousto-optic deflectors (AODs) for beam steering offer an alternative to traditional galvanometer scanners (Bullen *et al*. 1997; Iyer *et al*. 2003) or can be used to only replace resonant galvo (Lechleiter *et al*. 2002) for x-axis scanning, enabling higher lateral scanning speeds and improved temporal resolution. In the axial direction, mechanical methods such as piezo-driven objective lenses are typically slow for fast 3D volume imaging. To overcome this limitation, rapid z-axis focal plane adjustments can be achieved using AODs (Geiller *et al*. 2020; Reddy and Saggau 2005), spatial light modulator (SLM) (Dal Maschio *et al*. 2011) ETL (Grewe *et al*. 2011) or remote scanning mirrors (Botcherby *et al*. 2012).

Second, more effective scanning strategies can be employed to further enhance imaging speed. These approaches focus on optimizing the scanning path or regions of interest (Katona *et al*. 2012; Sofroniew *et al*. 2016; Stirman *et al*. 2016). Instead of the traditional raster scan method, arbitrary line scanning can be optimized to sample a large volume sparsely or targeted to specific regions or cells. For example, a heuristically optimal path was implemented scanning to record activity from thousands of neurons at 8.5 Hz (Sadovsky *et al*. 2011). Sofroniew *et al*. developed 2pRAM (two-photon random access mesoscope) achieving random access to multiple brain regions and providing diffraction-limited resolution in a cylindrical volume measuring 5 mm in diameter and 1 mm in depth (Sofroniew *et al*. 2016).

**Figure 4:**
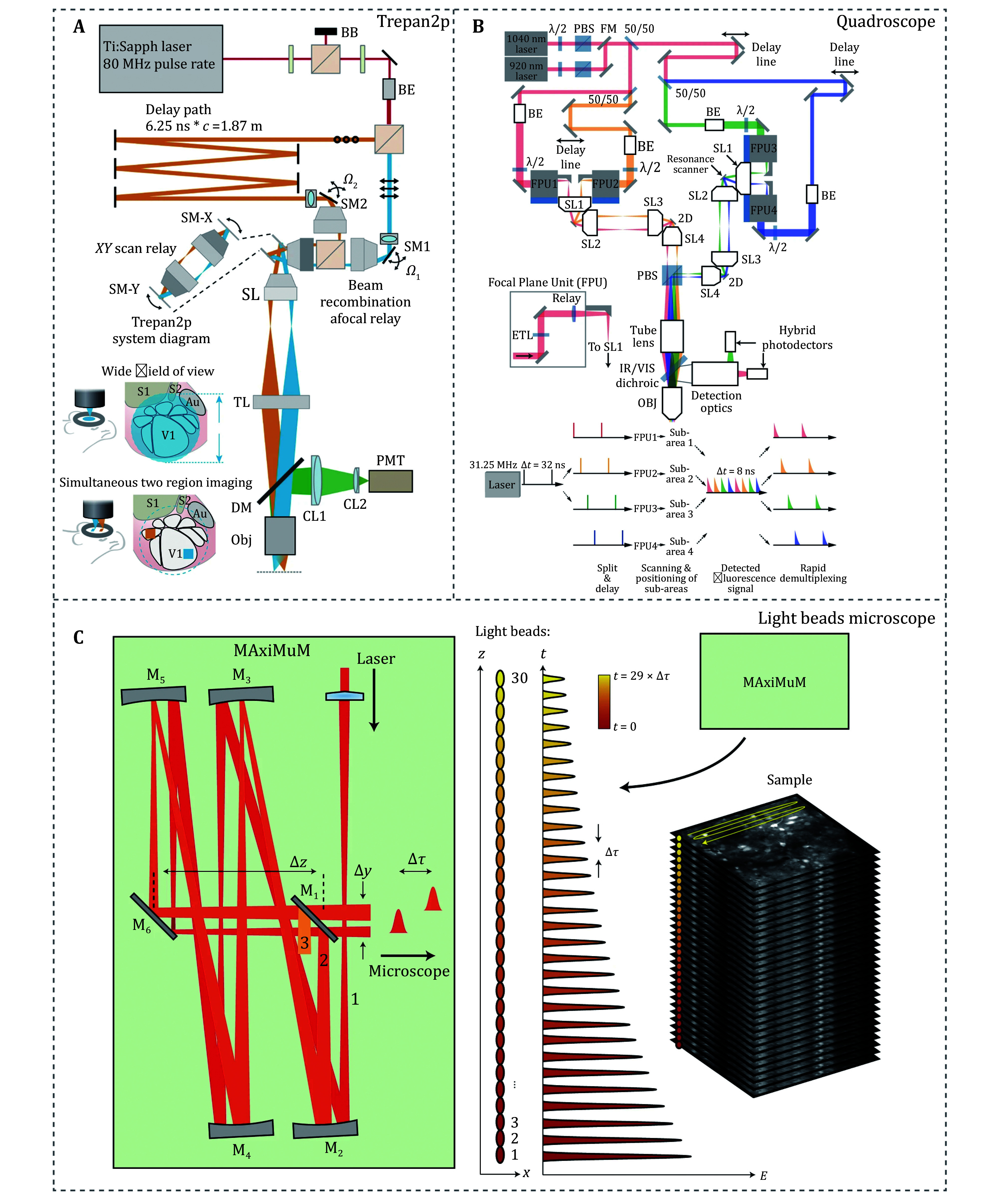
**A** Schematic of the Treapn2p system, enabling simultaneous imaging of two regions by temporal multiplexing (Stirman *et al.* 2016). **B** Schematic of the Quaroscope system, enabling simultaneous imaging of four regions by temporal multiplexing (Clough *et al.* 2021). **C** Schematic of light beads microscope system, enabling simultaneous imaging of 30 layers by temporal multiplexing (Demas *et al.* 2021)

Third, implementing multiple foci to parallelize imaging through multiplexing can increase the imaging speed by a factor proportional to the number of foci when imaging large scale regions or multiple planes (Fig. 4). One approach is to use multiple detectors (Lecoq *et* *al*. 2014) or a photon array detector (Vaziri and Shank 2010) to record signals simultaneously. Another strategy involves temporally multiplexed foci, where the optical path is adjusted to separate signals temporally from different focal regions between laser pulses (Amir *et al.* 2007; Cheng *et al.* 2011; Clough *et al.* 2021; Demas *et al.* 2021; Stirman *et al.* 2014, 2016; Yu *et al.* 2021). Stirman *et al*. designed temporally multiplexed excitation pathways to simultaneously record two regions, achieving an expanded field of view of approximately 10 mm^2^ (Stirman *et al*. 2016) (Fig. 4A). Clough *et al*. developed Quadroscope, which enables the recording of four regions at approximately 10 Hz, covering eight brain regions. Demas *et al*. developed the LBM (light beads mesoscope), splitting the ultrafast pump pulse into 30 copies, which are delayed in time and focused into different depths, enabling the recording of over a million neurons (Demas *et al*. 2021) (Fig. 4C) and providing comprehensive insights into how different brain regions respond to sensory inputs and the neural representations of working memory during behavioral learning, including their stability and causal relationships (Bellafard *et al*. 2024).

The heterogeneity of biological tissues in intravital imaging induces scattering and aberrations, which significantly affect laser scanning imaging. These effects can degrade resolution, reduce the SNR, and decrease excitation efficiency. The distortion of the light is able to be counteracted by adaptive optics (AO) techniques (Ji *et al*. 2010). An adaptive optics system typically consists of a wave front sensing module, which can directly measure aberrations using devices such as a Shack-Hartmann wave front sensor or a pyramid wave front sensor to detect the distorted wave front. Additionally, a wave front correction element, such as a deformable mirror or an SLM, is employed to compensate for the aberrations, thereby achieving optimal focusing for high-quality imaging (Hampson *et al*. 2021). For large FOV correction, Park *et al*. developed a multi-pupil adaptive optics system that maps separate regions of the sample onto different pupil locations (Park *et al*. 2017). Each region undergoes independent AO correction, effectively enabling large-scale, aberration-free intravital imaging across the entire field of view. Similarly, the 2pSAM (two-photon synthetic aperture microscope) achieves fast, aberration-free 3D imaging with low phototoxicity by utilizing separated apertures to capture images from different angles. These images are then digitally combined through a process known as digital adaptive optics (DAO) (Zhao *et al.* 2023). This approach enables high-speed volumetric imaging with minimal distortion, and reduced phototoxicity, making it well-suited for long-term intravital mesoscale imaging.

In summary, current laser scanning techniques, through the innovative design of objective lenses, scanning engines, scanning strategies, as well as the integration of multiplexing and adaptive optics technologies, have significantly enhanced the performance of intravital mesoscale imaging. Notably, many advanced imaging systems achieve superior performance by combining multiple complementary technologies, leading to substantial improvements in data throughput, SNR, resolution, and overall imaging quality, effectively addressing the challenges of large-scale, high-resolution intravital imaging.

### Computational imaging

With the development of computational techniques, an increasing number of methods can modulate either the illumination or detection paths to achieve coded intravital microscope imaging at the mesoscale. These approaches overcome optical limitations, enabling imaging systems to transmit and reconstruct more information beyond traditional constraints.

One key objective of computational imaging is tomography, as it enables rapid acquisition of 3D spatial information in living samples. Optical coherence tomography (OCT) is widely used for large-scale retinal imaging (Wojtkowski *et al*. 2005) in both animals and humans. Based on low-coherence interferometry, it measures the time delay and intensity of light reflected or scattered from different depths within the sample to obtain high-resolution 3D structures. Fluorescence laminar optical tomography (FLOT) at mesoscale employs laminar illumination to acquire fluorescence projection data from multiple angles (Hillman *et al*. 2007; Yuan *et al*. 2009). Through tomographic reconstruction algorithms, it calculates the 3D fluorescence distribution, enabling applications such as neural activity recording in 3D. Light field imaging techniques (Guo *et al*. 2019; Levoy *et al*. 2006) have been increasingly popular in recent years as a 3D tomographic approach due to their high spatial resolution, tomographic capability, and ability to achieve rapid 3D imaging with low phototoxicity (Orth and Crozier 2013; Scrofani *et al*. 2018; Wagner *et al*. 2019; Xue *et al*. 2020). They are commonly used to capture dynamic processes in living organisms, such as neural activity in zebrafish (Cong *et al*. 2017) and mouse and immune responses in the mouse liver or cortex (Nöbauer *et al.* 2017, 2023; Prevedel *et al.* 2014; Scrofani *et al.* 2018). Microlenses are implemented in the detection path of light field imaging, not only extending the DOF but also capturing both spatial 2D information and angular information simultaneously (Levoy *et al.* 2006; Xiong *et al.* 2021a). Zhang *et al*. developed a precise model for light-field imaging (Zhang *et al*. 2021a), effectively eliminating scattering backgrounds in living tissues, which enables the precise reconstruction of a 3D volume from a single snapshot through post-processing.

Another critical objective is to correct optical aberrations, addressing a century-old challenge in optical design through computational approaches. Traditional optical methods for mesoscale imaging face inherent limitations, primarily due to spatially non-uniform aberrations that arise from the high-throughput optical system design and fabrication process. Additionally, environmental aberrations and scattering induced by tissue heterogeneity further degrade resolution and SNR, while prolonged fluorescence excitation leads to phototoxic effects, limiting long-term high-speed observations. To address these challenges, Eric Betzig’s team introduced adaptive lattice light-sheet microscopy (Liu *et al.* 2018), which integrates hardware-based adaptive optics on both the illumination and detection paths. This technique effectively corrects aberrations within a small field of view (FOV) while reducing phototoxicity, enabling long-term imaging. However, this approach inherently compromises 3D imaging speed and the effective imaging FOV.

**Figure 5:**
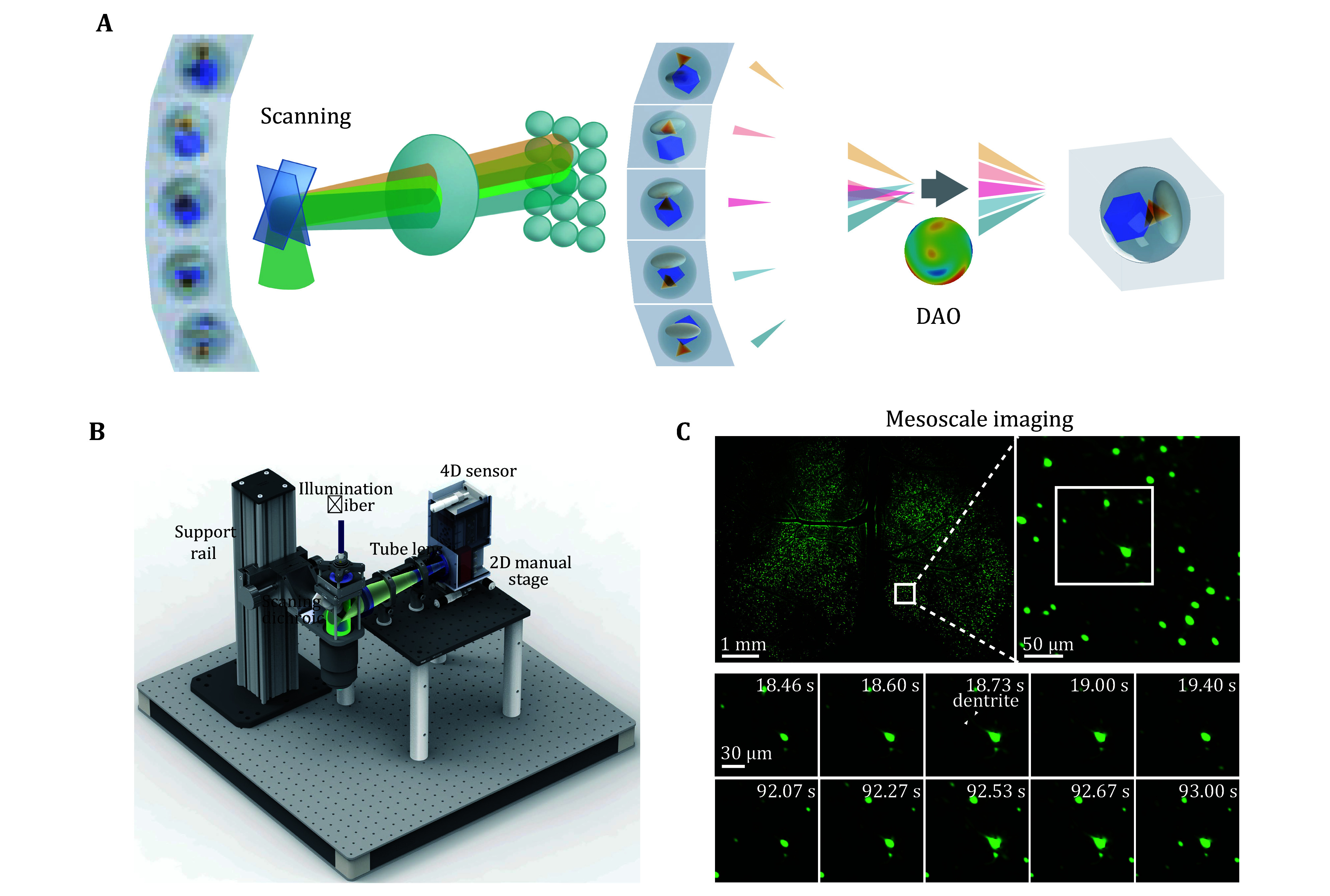
**A** Principle of scanning light field imaging mechanism and digital adaptive optics computational framework (Wu *et al.* 2021). **B** Design and assembly of RUSH3D system (Zhang *et al.* 2024a). **C** Mesoscale cortex imaging by RUSH3D system. Top, a perspective of the imaged mouse cortex with thousands of neurons. The FOV is across 8 mm × 6 mm × 400 μm. with a zoom-in panel. Bottom: Time-lapse visualization of the neuron of interest. As the neuron fires, the dendrite becomes clearly distinguishable (Zhang *et al.* 2024a)

To further address the challenge of optical aberrations, Wu *et al*. developed a computational imaging framework in 2021 (Wu *et al.* 2021) with detailed practical guidance in 2022 (Lu *et al.* 2022), introducing a high-dimensional spatiotemporal scanning mechanism (Fig. 5A). By leveraging aperture diffraction encoding to impose coherent constraints on an incoherent light field (Fig. 5A), they resolved the trade-off between spatial and angular resolution in conventional light-field imaging methods. This innovation established a novel paradigm of 3D excitation detection, enhancing intravital imaging spatiotemporal resolution by two orders of magnitude while reducing phototoxicity by three orders of magnitude. The imaging system achieved diffraction-limited resolution at millisecond-level speeds, extending the 3D imaging for intravital observation from the minute scale to six hours.

Further advancing computational imaging, Wu *et al*. proposed digital adaptive optics (DAO) for incoherent light imaging in 2022 (Wu *et al.* 2022). Unlike traditional AO, which relies on SLMs to manipulate wave fronts for aberration correction, DAO estimates aberrations from multi-view data and digitally redistributes light rays in post-processing. This decouples the aberration correction from the imaging process (Cao *et al.* 2024; Guo *et al.* 2024), achieving high-speed, large-scale aberration compensation and opening new avenues for solving the century-old challenge of optical aberrations.

By integrating these two computational imaging innovations, a high-speed, low-phototoxicity mesoscale intravital imaging system (RUSH3D) (Zhang *et al.* 2024a) capable of capturing dynamic 3D imaging across the entire cortical region of live mice at cellular resolution is developed. This system achieves rapid volumetric mesoscale imaging with a FOV of 8 mm × 6 mm × 0.4 mm at 20 Hz, enabling continuous intravital imaging for over 10 h with low phototoxicity (Figs. 5B and 5C). This advancement opens up a new horizon for the study of large-scale intercellular interactions at the mammalian organ level across a long term, which is critical for neural circuit mechanisms, tumor metastasis, and immune responses and may lead to data-driven large-scale biological investigations.

Further improvements were made by integrating a confocal module, which physically suppresses an out-of-focus background (Lu *et al.* 2025). Additionally, multi-angle imaging (Chen *et al.* 2024b; Xiong *et al.* 2021b) and the introduction of spherical aberration phase modulation (Zhang *et al.* 2022) were employed to improve axial resolution and expand the effective axial imaging range. Meanwhile, advancements in 3D reconstruction algorithms, including traditional iterative methods (Broxton *et al*. 2013; Lu *et al*. 2019) and machine learning approaches, have contributed to improved resolution (Liu *et al.* 2023a, 2023b; Lu *et al.* 2023; Wagner *et al.* 2019; Wang *et al.* 2021), artefact reduction (He *et al.* 2021; Zhang *et al.* 2021b; Zhu *et al.* 2022), and enhanced reconstruction speed (Wagner *et al.* 2021; Wang *et al.* 2021), making 3D intravital mesoscale imaging more accessible and efficient.

## DISCUSSION AND FUTURE PERSPECTIVES

Intravital mesoscopic imaging has made remarkable progress with the advancement of diverse imaging modalities, enabling high-resolution, large FOV visualization of dynamic biological processes. On this basis, it is essential to address the remaining challenges and explore potential directions for future development.

Although visualization of centimeter-scale tissues is achieved in *ex vivo* imaging, such as mapping an entire rhesus monkey brain (Xu *et al*. 2021) or a whole mouse body (Cai *et al*. 2023), achieving whole-brain neural activity recording intravitally remains challenging. This limitation arises from constraints in the SBP at video-rate temporal resolution and the restricted imaging depth in living tissues due to scattering and aberrations. Therefore, it is crucial to continue expanding the SBP for larger biological systems and to penetrate deeper into scattering tissues with minimally invasive surgical techniques. Achieving higher SBP requires not only improvements in optical design to maintain cellular resolution over centimeter-scale fields of view but also innovations in sensor technology and multiplexing strategies. The ability to capture detailed cellular activities across extensive brain regions or whole organs in larger and higher-order animals will profoundly enhance our understanding of complex biological networks (Gilman *et al*. 2017).

As for deeper imaging, future strategies will likely integrate novel modalities such as photoacoustic imaging, which achieves greater penetration depths with high resolution. Alternatively, multiphoton techniques, such as three-photon microscopy (Horton *et al*. 2013), or longer-wavelength fluorophores (Hong *et al*. 2017) in the near-infrared spectrum could be employed, as they offer enhanced tissue penetration, reaching depths of approximately 1 mm below the cortex (Wang *et al.* 2022; Zhao *et al.*, 2023). Additionally, advancements in implantable optics (Paraskevopoulos *et al*. 2022) and non-invasive window (Drew *et* *al*. 2010; Li *et al*. 2022) or other clearing techniques (Boothe *et al.* 2017; Ou *et al.* 2024) could further reduce tissue disruption, enabling long-term, deep-tissue imaging in living organisms.

A continued focus will be on translating optical bottlenecks into computational solutions. The synergy between optical hardware and computational algorithms will be pivotal in overcoming traditional optical limitations. Computational imaging techniques, such as light field reconstruction (Wagner *et al.* 2021; Wang *et al.* 2021; Wu *et al.* 2021; Zhu *et al.* 2023), compressed sensing (Pavillon and Smith 2016), and deep learning-based deconvolution (Qiao *et al*. 2021), can compensate for aberrations, enhance resolution, and recover high-fidelity 3D structures from mesoscale data. Future imaging systems will likely be co-designed with advanced computational frameworks (Zhang *et al.* 2023b), transforming raw optical signals into rich, interpretable biological information.

Besides, mesoscale imaging generates vast amounts of data, posing significant challenges in storage, processing, and analysis. Developing efficient data handling pipelines that integrate real-time processing and automated imaging enhancement will be challenging and essential (Li *et al.* 2023c). This includes large-scale neural signal extraction (Zhang *et al.* 2023c), high-throughput cell tracking (Arbelle *et al*. 2018), large-field image reconstruction (Zhang *et al.* 2024a) and super-resolution (Foylan 2024), denoising (Chen *et al.* 2024a; Li *et al.* 2021b, 2023a, 2023b; Qiao *et al.* 2024; Zhang *et al.* 2023a), color correction (Zhuang *et al.* 2021), motion drift correction (Pnevmatikakis and Giovannucci 2017), image domain transformation (Li *et al.* 2021a) and mesoscale neuronal data analysis (Cai *et al.* 2022; Xiao *et al.* 2024). Furthermore, the integration of artificial intelligence and machine learning algorithms will accelerate the interpretation of complex datasets, enabling the extraction of meaningful biological insights from terabytes of imaging data.

As mesoscale imaging technologies continue to evolve, their integration with computational methods and innovative imaging modalities will expand the boundaries of intravital biological research. By addressing the challenges of scale, depth, and data complexity, future imaging platforms will offer unprecedented insights into the dynamic processes that govern life at the cellular and biological systems level.
